# Medial prefrontal activation as a neural predictor of cognitive training gains in older adults with amnestic mild cognitive impairment: exploratory evidence

**DOI:** 10.3389/fnagi.2026.1733025

**Published:** 2026-02-04

**Authors:** Soowon Park, Yeeun Byeon, Jung-In Lim, Hyeonjin Kim, Jun-Young Lee

**Affiliations:** 1 Division of Teacher Education, College of Liberal Arts, Kyonggi University, Suwon, Republic of Korea; 2 Department of Medical Device Development, Seoul National University College of Medicine, Seoul, Republic of Korea; 3 Department of Psychiatry, Seoul Metropolitan Government-Seoul National University College Boramae Medical Center, Seoul, Republic of Korea; 4 Department of Psychiatry, Seoul National University of Medicine, Seoul, Republic of Korea

**Keywords:** aMCI, attentional task, fNIRS, medial prefrontal cortex, neural predictors, personalized education

## Abstract

**Background:**

Not all individuals benefit equally from cognitive training, making it essential to identify neural markers that can predict individual responsiveness, particularly among those at the early stages of cognitive impairment.

**Methods:**

This study investigated whether prefrontal activation measured by functional near-infrared spectroscopy (fNIRS) during three categories of training tasks (attentional, imagery, and associative) predicts cognitive gains in older adults. Twenty-two older adults with amnestic mild cognitive impairment (aMCI) completed a 12-week digital-device–based cognitive training program comprising 12 tasks (6 attentional, 3 imagery, 3 associative). Prefrontal activation was recorded via fNIRS during each task. Cognitive improvement was assessed using the Alzheimer's Disease Assessment Scale–Cognitive Subscale at baseline and 3 months (*n* = 22).

**Results:**

Among the three task categories, only attentional training was associated with prefrontal activation patterns that significantly predicted cognitive improvement at 3 months. Specifically, greater activation in the left mPFC during attentional training was negatively associated with ADAS-Cog scores (β = –.443, *p* = 0.039), indicating that individuals with higher activation exhibited greater cognitive improvement.

**Conclusion:**

The findings suggest that increased medial prefrontal activation during attentional training reflects compensatory engagement and task-related control processes, which in turn predict larger cognitive gains in individuals with aMCI. These results indicate that neural activation measured during training can serve as a reliable predictor of subsequent cognitive gains, offering a potential foundation for designing personalized and adaptive educational programs.

## 1 Introduction

Not all individuals benefit equally from cognitive training; some demonstrate substantial improvement, while others show little to no change despite receiving the same intervention (Bürki et al., 2014). Identifying objective indicators that can reliably predict an individual's enhancement from training is essential for personalized education and for optimizing the effectiveness of the intervention (Gozdas et al., 2024; Shani et al., 2019). Neural activation during training has emerged as a promising candidate predictor (Lee et al., 2023). Given that neural activation reflects the brain's real-time engagement with task demands and underlying information processing mechanisms, it provides a direct neurobiological window into the cognitive processes that mediate learning outcomes (Hu et al., 2019). Importantly, advances in non-invasive neuroimaging technologies, particularly functional near-infrared spectroscopy (fNIRS), enable objective quantification of neural activation, thereby offering a practical and reliable biomarker for predicting individual differences in training-induced gains (Acevedo et al., 2022; Nouchi et al., 2020).

Previous studies have demonstrated that prefrontal cortex activation is particularly linked to cognitive training tasks engaging attentional control and executive functions. Meta-analytic evidence indicates that a core prefrontal control network—including medial and lateral prefrontal regions—supports a broad range of executive processes such as selective and divided attention, working memory updating, and top-down regulation of cognitive resources (Niendam et al., 2012). Consistent with this framework, studies in aging populations have shown that tasks requiring sustained attention and executive control reliably engage prefrontal regions and are sensitive to individual differences in cognitive capacity (Voss et al., 2010).

Within fNIRS-based training research, prefrontal activation measured during active cognitive engagement has been shown to predict individual differences in training-related gains, particularly for cognitively demanding tasks that place high demands on attentional and executive control in both healthy older adults and individuals with mild cognitive impairment (Vermeij et al., 2017; Herold et al., 2018). Importantly, training-induced modulation of prefrontal activation has also been associated with broader indices of cognitive plasticity and reserve, suggesting that real-time neural responses during cognitively demanding tasks may reflect the capacity for subsequent cognitive improvement (Belleville et al., 2023; van Balkom et al., 2020). Together, these findings support the notion that prefrontal activation during training—especially during tasks engaging attentional and executive processes—may serve as a candidate neural marker for predicting individual variability in future cognitive gains.

Despite growing interest in neural predictors of training efficacy, previous research has predominantly examined changes in neural activation measured at pre- and post-training time points, with comparatively limited investigation into the predictive utility of activation patterns during training itself—particularly in relation to task-specific characteristics (Herold et al., 2018; van Balkom et al., 2020). Critically, not all training activities are likely to possess equivalent predictive validity, as distinct tasks impose varying cognitive demands and engage partially dissociable neural networks, which may differentially influence the reliability of brain activation as a predictor of subsequent gains (Just et al., 2001). This notion, however, remains largely speculative, underscoring the need for systematic empirical investigation to elucidate which task characteristics yield the most informative neural signatures.

Identifying the specific features of training tasks that generate neural activation patterns robustly associated with cognitive improvement holds both practical and theoretical significance, particularly in clinical populations at elevated risk for cognitive decline. Individuals with aMCI, who exhibit measurable memory deficits and face heightened risk of progression to dementia (Petersen, 2004), represent a critical target population for early intervention. In this context, the ability to predict training responsiveness becomes especially consequential, as timely identification of optimal training paradigms could maximize cognitive reserve during a pivotal window for intervention, potentially delaying or mitigating further deterioration (Stern et al., 2019). Theoretically, examining neural predictors in aMCI would illuminate not only the mechanisms underlying training-induced plasticity and transfer, but also the extent to which degenerative processes alter the brain's capacity to benefit from intervention, thereby informing our understanding of preserved vs. impaired learning mechanisms in pathological aging.

The present study explored whether prefrontal activation during three categories of training tasks—attentional, imagery/visuospatial, and associative—could serve as a predictor of subsequent cognitive gain, as measured by the Alzheimer's Disease Assessment Scale–Cognitive Subscale (ADAS-Cog). These domains were selected because they are among the cognitive functions most commonly affected in amnestic mild cognitive impairment and are closely related to everyday functional challenges in older adults (Gauthier et al., 2006).

Importantly, these task categories align with the core components of metamemory-based training, which emphasizes active monitoring and strategic control of cognitive processes rather than simple repetition (Park et al., 2018). Prior work on multi-strategic memory training suggests that attentional control, imagery-based strategies, and associative processing are central mechanisms through which cognitive improvement is achieved, as these processes engage executive and prefrontal control systems. Within this framework, attentional processes were expected to exhibit particularly strong predictive value, given their foundational role in early-stage cognitive operations and their influence on downstream memory encoding and retrieval (Savulich et al., 2019).

## 2 Methods

### 2.1 Participants

A total of 22 community-dwelling older adults, aged 55 to 85 years, who reported subjective memory difficulties, were recruited through the Department of Psychiatry at Boramae Medical Center. The diagnosis of aMCI was determined using established international standards, including the DSM-IV (The Diagnostic and Statistical Manual of Mental Disorders, Fourth Edition) diagnostic framework, the NINCDS–ADRDA criteria for Alzheimer's disease (McKhann et al., 1984) and Petersen's criteria for MCI (Petersen et al., 1997). All participants met the amnestic subtype specification.

Inclusion criteria required a Clinical Dementia Rating (CDR) score between 0.5 and 1.0, along with performance at least 1.0 standard deviation below age-adjusted norms on one or more memory subtests of the Korean Version of the Consortium to Establish a Registry for Alzheimer's Disease neuropsychological battery (CERAD-K) (Lee et al., 2002). To ensure feasibility of the smartphone-based cognitive training program, participants were required to have adequate vision, hearing, and speech abilities, as well as basic smartphone proficiency. These criteria were assessed based on participants' current smartphone use and a brief screening process that incorporated self-report confirmation from a caregiver or family member. Exclusion criteria included the presence of medical or psychiatric conditions likely to interfere with participation, or recent initiation of cholinergic or non-cholinergic treatments that could affect cognition. Demographic and clinical characteristics of the sample are presented in Table 1.

**Table 1:** Baseline sociodemographic and neuropsychological characteristics of participants.

**Variables**	**Participants with aMCI (*N* = 22)**
**Sociodemographics**	
**Age (years)**	
Mean (standard deviation)	72.95 (7.15)
Range	58-82
**Gender**, ***n*** **(%)**	
Male	7 (32)
Female	15 (68)
**Education (years)**	
Mean (standard deviation)	11.50 (5.50)
[1] No education (0 year)	1 (5)^a^
[2] Elementary school (1-6 years)	3 (14)
[3] Middle school (7-9 years)	4 (18)
[4] High school (10-12 years)	4 (18)
[5] University (13 ≤ years)	10 (45)
**Neuropsychological measures**	
**MMSE score**	
Mean (standard deviation)	24.96 (3.60)
Range	19-30
CDR global score	22(100)^a^

^a^ n (%).

aMCI, amnestic mild cognitive impairment; MMSE, mini-mental state examination; CDR, clinical dementia rating.

### 2.2 Cognitive training

Cognitive training was implemented by administrating Cogthera (Emocog Inc.), a smartphone-based digital cognitive therapeutic program in South Korea (Byeon and Lee, 2025). The program was implemented over 12 weeks and included a total of 12 structured tasks designed to train core domains of cognition. Six tasks targeted attentional control (e.g., selective and divided attention), three tasks emphasized imagination (e.g., visuospatial imagination), and three tasks focused on associative memory (e.g., paired-association and memory linking). Task difficulty was automatically adjusted according to individual performance, thereby ensuring adaptive progression and sustained engagement throughout the training.

### 2.3 Procedures

**Figure 1:**
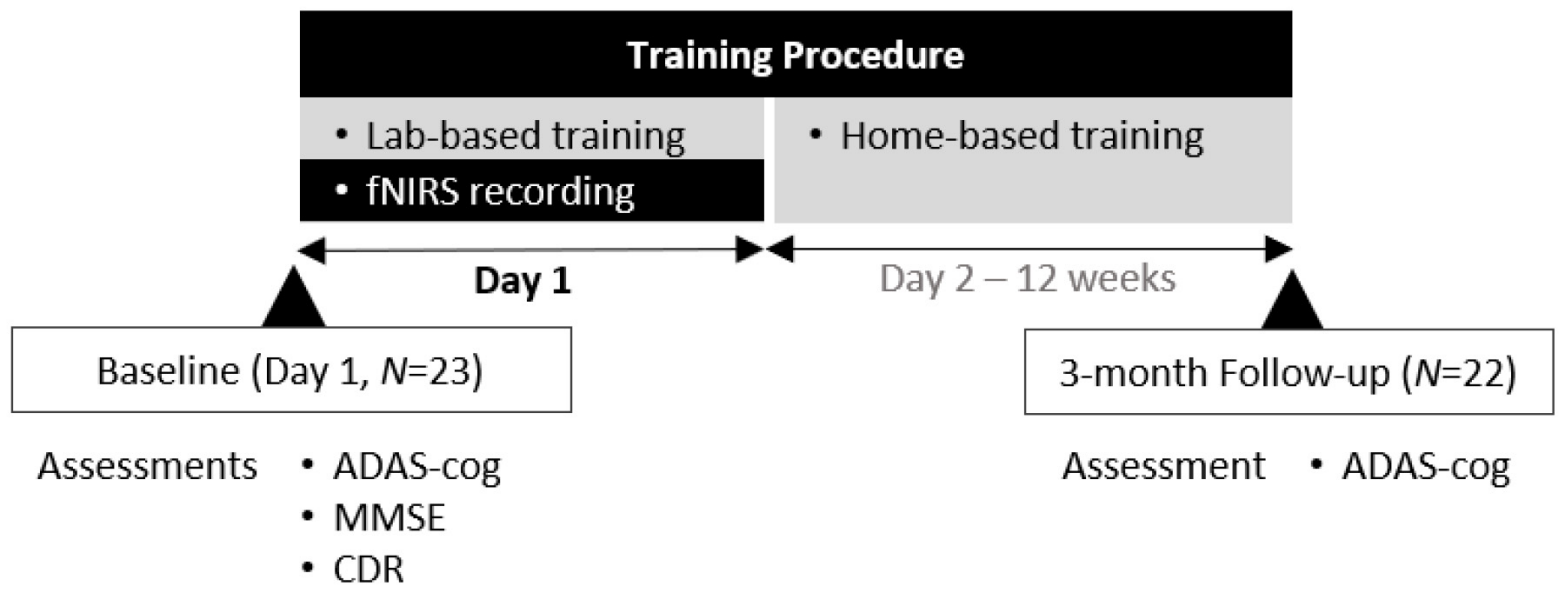
Overview of the study procedure.

The overall study procedure is illustrated in Figure 1. At baseline (Day 1), all participants (*N* = 23) completed a cognitive assessment using the ADAS-Cog, Mini-Mental State Examination (MMSE), and Clinical Dementia Rating (CDR) prior to the initiation of training. On the same day, participants engaged in a laboratory-based training session, during which prefrontal activation was recorded with fNIRS. fNIRS data were collected only during the initial, lab-based training session on Day 1. No neuroimaging measurements were obtained during the subsequent home-based training sessions conducted over the 12-week period. Following this initial session, participants continued with a structured 12-week home-based cognitive training program (Day 2 to Week 12). During this period, participants were instructed to engage in the smartphone-based training freely on a daily basis, with up to two training sessions per day. Training adherence was monitored throughout the intervention period, and participants who did not engage in training received reminder notifications via smartphone pop-up messages and follow-up phone calls. Cognitive outcomes were evaluated at 3 months, at which point 22 participants were reassessed using the ADAS-Cog to determine immediate post-training effects. One participant did not complete the follow-up assessment due to personal reasons and chose to withdraw from further participation. Accordingly, cognitive performance was assessed at two time points: baseline (Day 1) and follow-up (3 months). fNIRS data were acquired exclusively during the baseline laboratory training session to examine whether neural activation during initial training predicted subsequent cognitive gains.

### 2.4 Measurements

#### 2.4.1 Korean version of the Alzheimer's disease assessment scale–cognitive subscale (ADAS-Cog)

Cognitive function was measured using the Korean version of the ADAS-Cog (Suh and Mohs, 2003). The Korean version of ADAS-Cog was developed and standardized to assess cognitive decline and behavioral symptoms in Korean patients with dementia. The ADAS-cog comprises 11 items evaluating memory (immediate word recall, orientation, word recognition, and recall of test instructions), language (object and finger naming, word-finding difficulty, following verbal commands, spoken language ability, and comprehension of spoken language), and praxis (constructional and ideational praxis). Scores range from 0 to 70, with higher scores indicating greater impairment.

#### 2.4.2 Mini-mental state examination (MMSE)

Global cognitive function was assessed with the Korean version of the Mini-Mental State Examination (K-MMSE), a standardized screening tool for cognitive impairment originally developed by Folstein et al. (1975) and validated for use in Korean older adults (Lee et al., 2002). The K-MMSE provides a total score ranging from 0 to 30, with higher scores indicating better cognition. It consists of seven components: orientation to time and place (10 points), registration (3 points), recall (3 points), attention and calculation (5 points), repetition (1 point), language (2 points), and performance of complex commands (6 points).

#### 2.4.3 Clinical dementia rating (CDR)

Dementia severity was further evaluated using the Clinical Dementia Rating (CDR) scale (Hughes et al., 1982; Morris, 1993), which was adapted and validated for Korean populations by Choi et al. (2001). The CDR assesses six functional domains—memory, orientation, judgment and problem solving, community affairs, home and hobbies, and personal care—and integrates these ratings into a global composite score. The global score indicates five levels of cognitive impairment: 0 (none), 0.5 (questionable), 1 (mild), 2 (moderate), and 3 (severe).

### 2.5 fNIRS data acquisition

Brain activity was measured with a portable fNIRS device (NIRSIT, OBELAB Inc., Korea). The system comprised 24 emitters and 32 detectors, producing 48 measurement channels in total. The source–detector separation was maintained at approximately 3 cm, and data were continuously sampled at rate of 8.138 Hz (OBELAB Inc., 2022).

All recordings were performed with participants seated in a stable and comfortable position. During the resting-state baseline session, they were instructed to keep their eyes closed, remain awake, and avoid engaging in intentional thought. Previous fNIRS studies have employed baseline recordings ranging from 2 s to 10 min (Herold et al., 2018). Based on the previous study, the baseline of the current study was recorded for 5 min.

After the baseline recording, participants watched a tutorial video embedded in the training application to ensure they understood how to operate the program. Once familiarized, they proceeded to complete the cognitive training tasks provided by the application. Task order was fully randomized at the individual participant level. Multiple randomized task-order sets were generated using a randomization table, and each participant was assigned a unique sequence in which the order of all cognitive training tasks was varied. This procedure ensured full counterbalancing across participants and minimized potential order effects and systematic bias related to task sequencing (Pollatsek and Well, 1995).

The hemodynamic response to task stimuli typically requires several seconds to reach its peak (around 6 s on average) (Orihuela-Espina et al., 2010; Pinti et al., 2018; Tong and de Frederick, 2010), and generally returns to baseline within 10–16 s following stimulus offset (Friston et al., 1998; Villringer and Dirnagl, 1997). To prevent overlap between successive trials, an inter-task interval of at least 30 s was provided.

**Figure 2:**
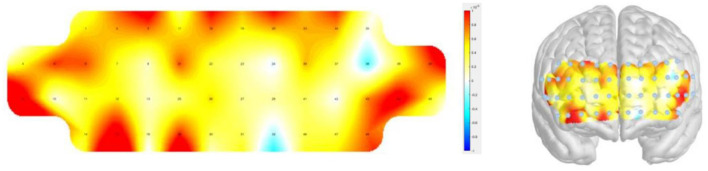
Activation map during the cognitive training. Left, 2D; Right, 3D.

Signal acquisition and preprocessing were carried out using the NIRSIT PC Tool (v.2.8) and NIRSIT Analysis Tool (v.3.7.5, OBELAB). Raw optical density signals were converted into concentrations of oxyhemoglobin (HbO) and deoxyhemoglobin (HbR) via the modified Beer–Lambert law (Badiola et al., 1993). All statistical analyses in the present study were conducted using HbO signals only, as HbO has been shown to be more sensitive to task-related cortical activation and to exhibit a higher signal-to-noise ratio than HbR in fNIRS research (Herold et al., 2018). Motion-related and systemic physiological noise was reduced by applying a discrete cosine transform (DCT) filter with a frequency range of 0.005–0.100 Hz. Data channels were excluded if more than 5% of frames were missing, if negative values exceeded 5% of the dataset, or if the signal-to-noise ratio (SNR) was below 30 dB. Any excluded channels were interpolated using a hierarchical padding approach, which involved replacing them with backup channels, averaging channels located in the same Brodmann area, or imputing with the global mean. A representative activation map obtained during the training session is displayed in Figure 2.

Prefrontal regions of interest (ROIs) were defined a priori based on standardized anatomical labeling and MNI-coordinate–based channel mapping provided by the device manufacturer (OBELAB Inc., 2022) and the official NIRSIT documentation. Four bilateral ROIs were specified: medial prefrontal cortex (mPFC), dorsolateral prefrontal cortex (dlPFC), ventrolateral prefrontal cortex (vlPFC), and orbitofrontal cortex (OFC). Channel distribution map of the NIRSIT system was presented in the Supplementary Figure 1 and Table 1 showed estimated MNI coordinates and anatomical labels for each fNIRS channel (OBELAB Inc., 2022).

### 2.6 Ethical considerations

Written informed consent was obtained from all participants prior to enrollment. The study protocol was reviewed and approved by the Institutional Review Board of Boramae Medical Center (IRB No. 20-2022-48) and was carried out in accordance with relevant ethical standards and regulations.

### 2.7 Statistical analysis

All statistical analyses were conducted using SPSS (version 20.0, IBM Corp., Armonk, NY, USA). The primary outcome variables were change scores in cognitive performance derived from the ADAS-Cog. Cognitive change was calculated by subtracting the baseline score from the follow-up score (Follow-up score – Baseline score), meaning that lower values reflected greater cognitive improvement.

Descriptive statistics were first computed for all study variables. Pearson's correlation analyses were performed to examine associations among demographic variables and cognitive outcomes. Paired-sample *t* tests were applied to compare ADAS-Cog scores between baseline and Follow-up (3 months).

To identify predictors of cognitive improvement, stepwise multiple regression analysis was conducted separately for cognitive change score. Predictor variables included demographic characteristics (age, gender, education) and mean fNIRS activation levels in predefined prefrontal regions of interest: the dorsolateral prefrontal cortex (dlPFC), ventrolateral prefrontal cortex (vlPFC), medial prefrontal cortex (mPFC), and orbitofrontal cortex (OFC), assessed bilaterally across the three task categories (attentional, imagery, associative). The threshold for statistical significance was set at *p* < 0.05 (two-tailed).

## 3 Results

### 3.1 Correlations between ADAS-Cog scores and demographic characteristics

Pearson's correlations were conducted to examine the associations between demographic variables (age, gender, education) and ADAS-Cog performance at each time point, as well as change scores. At baseline, older age was significantly associated with higher ADAS-Cog scores, *r* = 0.43, *p* = 0.044, indicating greater impairment. In contrast, higher years of education were significantly related to lower ADAS-Cog scores at baseline, *r* = −0.46, *p* = 0.030, suggesting better cognitive performance. Gender was not significantly correlated with baseline scores. At the 3-month follow-ups, none of the demographic variables were significantly correlated with ADAS-Cog performance. No significant associations were found between demographic variables and cognitive change scores.

### 3.2 Changes in cognitive performance from baseline to follow-up

Descriptive statistics and paired-sample *t* tests for ADAS-Cog scores are presented in Table 2. A decrease in scores was observed from baseline (*M* = 29.04, *SD* = 9.03) to the 3-month follow-up (*M* = 28.59, *SD* = 8.00), reflecting variability in individual change rather than a significant group-level improvement; the mean change was not statistically significant, *t*_(21)_ = 0.56, *p* =.58.

**Table 2:** Descriptive statistics and paired *t* tests for Alzheimer's disease assessment scale–cognitive (ADAS-Cog) scores.

**Time point**	***M*(*SD*)**	***SE***	***M_*dif*_***	**CI of *M_*dif*_***	***t***	***p***
Baseline	29.04(9.03)	1.92	0.45	[−1.23, 2.13]	0.563	.580
3-month	28.59(8.00)	1.71				

M, mean; SD, standard deviation; SE, standard error; Mdif, mean difference; CI, confidence interval.

To illustrate inter-individual variability in cognitive trajectories, Supplementary Figure 2 presents ADAS-Cog scores for each participant at baseline and 3-month assessments. The figure highlights marked heterogeneity, with some participants showing decreased scores (improvement), others remaining stable, and some exhibiting increased scores (decline).

### 3.3 Neural predictors of cognitive improvements

Stepwise multiple regression analysis including brain activation across three categories of training tasks (attentional, imagery, and associative) indicated that only activation of the left medial prefrontal cortex (mPFC) during attentional tasks significantly predicted cognitive improvement at the 3-month follow-up (β = −0.443; *p* = 0.036; adjusted *R*^2^ = 0.156; *F*_(1, 20)_ = 4.881; *p* = 0.039; Table 3). Greater activation in the left mPFC during attentional training was associated with larger cognitive gains.

**Table 3:** Stepwise multiple regression analysis of brain activity as a predictor of cognitive gain.

**Brain activity during cognitive training**	**B**	**SE**	**β**	***t***	***p***
Left medial prefrontal cortex during attentional task	−0.607	0.745	−0.443	2.209	0.039

## 4 Discussion

The present study provides exploratory evidence suggesting that neural activation measured during an initial cognitive training session may be related to individual differences in subsequent cognitive change among older adults with aMCI. By focusing on neural activation captured during training, rather than solely on pre- to post-training change, the findings highlight the potential prognostic value of real-time neural responses as candidate indicators of variability in training responsiveness. While these findings are necessarily tentative and should be interpreted cautiously given the limited sample size and analytic scope, they point to the potential relevance of prefrontal activation during attentional training for understanding heterogeneity in cognitive training outcomes. Importantly, these observations were derived from a sample of community-dwelling older adults with aMCI, underscoring the relevance of this exploratory approach for populations at elevated risk for cognitive decline.

The present findings suggest that activation of the mPFC during training can serve as a neural marker for predicting future changes in cognitive function. The mPFC is a central hub that is functionally embedded in both the default mode network (DMN) and the frontoparietal control network (FPCN), two large-scale systems that are critically involved in memory, executive processes, and attentional control (Dixon et al., 2018; Xu et al., 2016). Importantly, the mPFC is among the earliest regions to show disruption in the course of Alzheimer's disease, yet it also appears capable of compensatory plasticity in individuals at risk, such as those with aMCI (Gozdas et al., 2024). Thus, heightened mPFC activation during training may reflect compensatory engagement that facilitates subsequent cognitive resilience. In the context of cognitive training, mPFC engagement is particularly associated with attentional control, internally directed attention, and executive monitoring processes that support the strategic regulation of cognitive resources (Garrison et al., 2021). Accordingly, heightened mPFC activation during training may reflect compensatory recruitment of attentional and executive control mechanisms, facilitating more efficient monitoring and allocation of cognitive effort.

The observed association between mPFC activation during attentional training and subsequent cognitive improvement, as indexed by change in ADAS-Cog total scores, may be understood in light of the compensatory recruitment hypothesis, which proposes that heightened prefrontal engagement enables older adults to counteract normative declines in neural efficiency (Vermeij et al., 2017). Individuals who recruited the mPFC more strongly during demanding attentional tasks may have been better able to flexibly allocate cognitive resources, thereby supporting greater cognitive gains. Supporting this interpretation, prior evidence indicates that the mPFC plays an integrative role in sustaining attention and exerting top-down control, processes that facilitate strategic information selection and cognitive flexibility in aging populations (Jobson et al., 2021). Moreover, disruptions in mPFC connectivity with sensory regions have been linked to impaired suppression of irrelevant information and heightened distractibility, further underscoring the region's compensatory significance for attentional regulation in aging (Chadick et al., 2014).

These findings also converge with the Compensation-Related Utilization of Neural Circuits Hypothesis (Reuter-Lorenz and Cappell, 2008), which posits that older adults compensate for reduced neural efficiency by recruiting additional prefrontal resources, particularly under higher task demands. Collectively, these findings suggest that greater mPFC activation during training may index an individual's capacity for compensatory neural recruitment, which in turn predicts the magnitude of cognitive benefit derived from intervention. This interpretation underscores the potential of task-related prefrontal engagement as a neurobiological marker of training responsiveness in aging populations at risk for cognitive decline.

Importantly, the predictive effect emerged only for attentional, and not imagery or associative, training tasks. This task specificity underscores the foundational role of attentional control as a gateway for broader cognitive enhancement, as attentional processes are required for the successful execution of higher-order cognitive operations (Luchicchi et al., 2016). Prior evidence further demonstrates that attentional engagement and prefrontal activation contribute to both near and far transfer effects, reinforcing attention as a primary driver of cognitive reserve (da Silva et al., 2022; van Balkom et al., 2020).

## 5 Limitations and future directions

Several limitations should be acknowledged. First, the modest sample size (*N* = 22) limited statistical power and constrains the generalizability of the findings. Accordingly, the present results should be interpreted as exploratory and hypothesis-generating rather than as definitive evidence of robust effects. Future studies with larger, adequately powered samples are needed to confirm the stability and reproducibility of the identified neural predictors. Second, cognitive gains were operationalized as pre–post differences in ADAS-Cog scores. Although this approach provides an intuitive and easily interpretable index of overall cognitive change, it may be influenced by baseline performance, ceiling or floor effects, and regression to the mean. Given the exploratory nature of the present study and the modest sample size, this operationalization was adopted as a pragmatic first step. Future studies should apply analytic approaches that explicitly account for baseline differences to refine estimates of training-related change. Third, the current study focused solely on the initial state of training. However, there is a plausible critical window within the 12-week training period during which neural activation changes may more strongly predict future cognitive improvements. Future research should explore dynamic neural markers throughout the entire training duration to identify such windows and better understand the temporal dynamics of cognitive gains. Finally, the present analyses involved the examination of multiple candidate neural predictors relative to a modest sample size, and no formal correction for multiple comparisons was applied. As a result, the observed significant association may be susceptible to inflated Type I error and should be interpreted with caution. In particular, the possibility that the identified effect reflects a spurious finding cannot be ruled out. Independent replication in larger samples with preregistered analytic plans and appropriate correction for multiple testing will be essential to establish the robustness of the present findings.

## 6 Conclusion

Greater medial prefrontal activation during attentional training predicted cognitive gains, suggesting compensatory network recruitment in older adults with mild cognitive decline. These findings emphasize the regional and task specificity of neural predictors and highlight the central role of attentional control in supporting near-term plasticity. More broadly, real-time, task-embedded neural activation measures represent a promising tool for personalizing cognitive interventions. Assessing neural responses during training, rather than only at baseline or post-intervention, enhances ecological validity and predictive precision, and may ultimately enable adaptive tailoring of interventions to maximize individual benefit.

## Data Availability

The raw data supporting the conclusions of this article will be made available by the authors, without undue reservation.
