# Supplementary material for: Medial prefrontal activation as a neural predictor of cognitive training gains in older adults with amnestic mild cognitive impairment: exploratory evidence
Source: Front Aging Neurosci. 2026 Feb 4;18:1733025. doi: 10.3389/fnagi.2026.1733025 (PMC12913516; doi:10.3389/fnagi.2026.1733025)

**Supplementary Figure S1**

Channel distribution map of the NIRSIT system. Channels corresponding to the medial prefrontal cortex (mPFC), dorsolateral prefrontal cortex (dlPFC), ventrolateral prefrontal cortex (vlPFC), and orbitofrontal cortex (OFC) were identified based on MNI coordinates and anatomical labeling provided by the manufacturer. Channel-to-region mapping was adapted from the official NIRSIT documentation. Figure adapted with permission from OBELAB Inc. (2022).
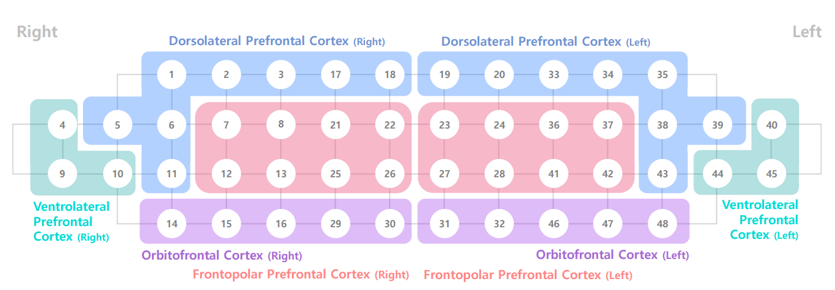


**Supplementary Figure S2**

Individual trajectories of Alzheimer’s Disease Assessment Scale–Cognitive (ADAS-Cog) scores at baseline and 3-month follow-up. Each line represents one participant. Lower ADAS-Cog scores indicate better cognitive performance. The figure demonstrates inter-individual variability in training outcomes.

*
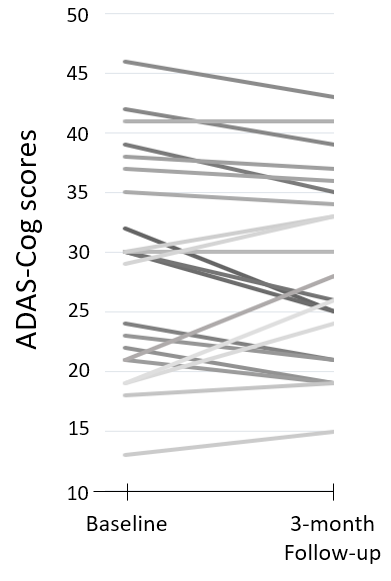
*

**Supplementary Table S1**

Estimated MNI coordinates and anatomical labels for each fNIRS channel. Channel-wise MNI coordinates (X, Y, Z), cortical location, and corresponding Brodmann area (BA) labels were provided by the device manufacturer and used for channel-to-region mapping in the present study. Table adapted with permission from OBELAB Inc. (2022).


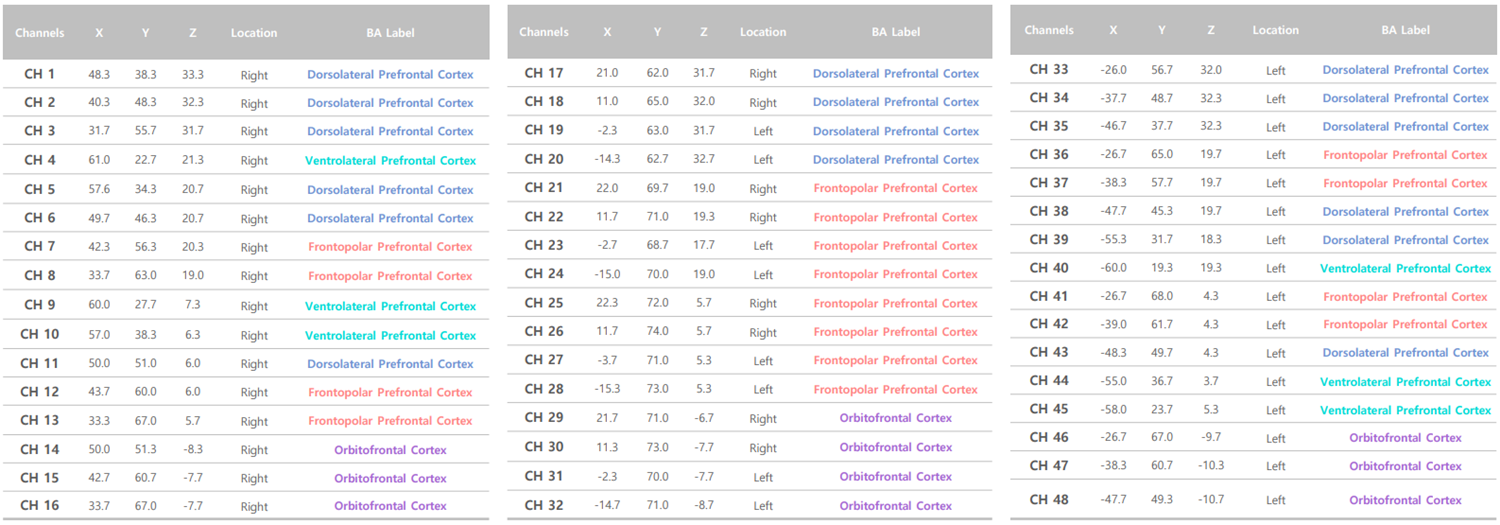

Supplement: Supplementary file 1 [file Data_Sheet_1.docx]
